# Surgical Management of Oligometastatic Non-Small Cell Lung Cancer

**DOI:** 10.3390/cancers17122040

**Published:** 2025-06-18

**Authors:** Susana Fortich, Deniz Piyadeoglu, Nafiye Busra Celik, Mara Antonoff

**Affiliations:** 1 Department of Surgery, University of Texas Medical Branch, Galveston, TX 77555, USA; sufortic@utmb.edu; 2 Department of Surgery, Division of Thoracic and Cardiovascular Surgery, MD Anderson Cancer Center, Houston, TX 77030, USA; piyadeogludeniz@gmail.com; 3 Department of Surgery, Division of Cardiac Surgery, Yale University School of Medicine, New Haven, CT 06510, USA; nbusracelik@hotmail.com

**Keywords:** oligometastatic non-small cell lung cancer, surgical resection, local consolidative therapy

## Abstract

**Background:** Oligometastatic non-small cell lung cancer (NSCLC) represents a biologically and clinically distinct state characterized by limited metastatic spread. Increasing evidence suggests that aggressive local therapies, including surgical resection, may confer a survival benefit in this population. The objective of this review is to evaluate the current role of surgery in the management of oligometastatic NSCLC, with emphasis on patient selection, surgical strategy, integration with systemic therapy, and ongoing clinical investigations. **Methods:** This narrative review synthesizes retrospective and prospective clinical data, meta-analyses, major consensus guidelines, and ongoing trials since 2012. We highlight prognostic factors, staging strategies, and the evolving role of molecular and biomarker-based stratification. **Results:** Multiple retrospective studies and several randomized trials have demonstrated improved progression-free and overall survival with local consolidative therapy in oligometastatic NSCLC. Prognostic factors associated with favorable outcomes include a limited number of metastases (≤3), good performance status, absence of mediastinal nodal disease, metachronous presentation, and actionable molecular alterations. The integration of surgery with systemic therapies, including targeted agents and immunotherapy, has become increasingly common in selected patients. Ongoing trials such as LONESTAR, NORTHSTAR, and BRIGHTSTAR are expected to further define the role of surgery in this setting. **Conclusions:** Surgery is emerging as a key component of multimodal treatment for carefully selected patients with oligometastatic NSCLC. Future efforts should focus on refining patient selection through molecular stratification and expanding prospective trial data to guide personalized biology-driven treatment strategies.

## Simple Summary

Historically, metastatic lung cancer has been treated with systemic therapy alone for life prolongation, with local therapeutic options considered for palliation of local symptoms. However, recent research demonstrates that local therapy consisting of radiation or surgery may improve survival. The majority of data in this space surrounds oligometastatic disease, meaning stage IV lung cancer that has spread to a limited number of sites and may benefit from more aggressive therapy. In this review, we explore the role of surgery for patients with limited metastatic lung cancer, including how medical teams choose the right patients, what surgical approaches are used, and how surgery fits with chemotherapy, targeted drugs, and immunotherapy. We also discuss important ongoing clinical trials and how future research may utilize molecular tests and blood-based markers to inform treatment decisions. Our goal is to help define when and how surgery could be used to improve outcomes for patients with oligometastatic lung cancer.

## 1. Introduction

Lung cancer remains the leading cause of cancer-related mortality worldwide, with non-small cell lung cancer (NSCLC) accounting for approximately 85% of cases. Approximately 340 people die each day from lung cancer, nearly 2.5 times more than the number of people who die from colorectal carcinoma [1]. Historically, the presence of metastatic disease in NSCLC has been considered a contraindication to surgical intervention, with systemic therapy being the primary treatment modality [2]. However, growing evidence has challenged this previous statement, in the setting of oligometastatic disease (OMD), a state characterized by limited metastatic burden that may offer potential for long-term survival or cure with aggressive local therapies [3,4]. Recent advances in molecular therapy have further improved patient selection, underscoring the importance of identifying actionable oncogenic driver mutations. As emphasized by the 2024 National Comprehensive Cancer Network (NCCN) guidelines, “Molecular testing for somatic, disease-associated oncogenic driver mutations or alterations should be conducted as part of broad molecular profiling, which is strongly recommended by the panel.” This molecular characterization has become critical in guiding personalized treatment strategies for patients with advanced and oligometastatic NSCLC [5].

The concept of oligometastases, first proposed in the 1990s, has gained increasing clinical relevance with advances in imaging, systemic therapies, and surgical techniques [6]. Oligometastasis refers to the concept of an intermediate state of cancer spread between localized disease and widespread metastases [7]. The significance of the oligometastatic state lies in its potential for curative-intent treatment, which contrasts with the historical palliative approach for metastatic NSCLC. Even as our most aggressive therapeutic strategies increasingly incorporate local consolidative therapy (LCT) for more widespread polymetastatic disease, OMD offers a more practical and achievable opportunity for comprehensive local consolidative therapy (cLCT), targeting all known sites of the disease at diagnosis. OMD thus represents a distinct clinical entity, different from both early-stage localized disease and more advanced polymetastatic spread. For carefully selected patients, aggressive local therapies, including surgical resection of both the primary lung tumor and metastatic lesions, have emerged as promising strategies to improve survival outcomes. 

When offered to well-vetted appropriate patients, surgical resection of the primary lung tumor and metastatic lesions has emerged as a promising therapeutic strategy. Multiple retrospective studies and, more recently, prospective data suggest that a multidisciplinary approach incorporating surgery can improve survival outcomes in select patients with oligometastatic NSCLC [3,8,9,10,11,12]. Recent meta-analyses have provided impressive evidence supporting the role of surgery in managing oligometastatic NSCLC. Zhang et al. analyzed three randomized controlled trials and five cohort studies that included 499 patients and demonstrated that local aggressive thoracic therapy, including surgery, particularly when administered after systemic therapy, reduced the risk of death by 47% and significantly improved survival outcomes. Similarly, another study presented better survival for surgical interventions with careful patient selection [11,13].

In acknowledgement of these advances, the Society of Thoracic Surgeons (STS) published a clinical practice guideline on the management of oligometastatic NSCLC in January 2025. These guidelines emphasize patient selection, the role of local therapies, including surgery, and the need for coordinated individualized care. Clear data exist to support the use of surgical resection of the primary lung tumor as local consolidative therapy for stage IV lung cancer [14].

This narrative review examines the current role of surgery in the management of oligometastatic NSCLC, with particular emphasis on the patient selection criteria, surgical strategies, integration of multimodal therapy, and future directions considering the 2025 STS guidelines. By synthesizing recent evidence and expert recommendations, we seek to clarify the evolving role of surgery in this complex patient population. However, as a narrative review, this work does not follow a formal systematic methodology, such as predefined MeSH terms, inclusion/exclusion criteria, or quality assessment tools. This may introduce selection bias and limit the reproducibility of our findings, which should be interpreted accordingly.

## 2. Biology and Classification of Oligometastatic NSCLC

### 2.1. Underlying Tumor Biology

The oligometastatic state is believed to represent a distinct biological phenotype characterized by limited metastatic potential. Unlike widely disseminated disease, oligometastatic tumors may exhibit genetic or epigenetic profiles that restrict their ability to colonize distant organs [15]. Molecular studies suggest that tumors in the oligometastatic state often show lower expression of genes associated with epithelial–mesenchymal transition (EMT), invasion, and immune evasion. This is supported by research indicating that oligometastatic tumors exhibit distinct biological characteristics compared to widely metastatic tumors. Specifically, these tumors tend to have a lower expression of EMT-related genes, which are crucial for the transition of epithelial cells to a more mesenchymal invasive phenotype. Additionally, oligometastatic tumors often show reduced expression of the genes involved in invasion and immune evasion mechanisms. For example, Katipally et al. (2023) discuss the role of epigenetic regulation in EMT and suggest that oligometastatic tumors may have a more epithelial phenotype with lower EMT activity, contributing to their limited metastatic potential [16]. Similarly, Ottaiano et al. (2023) highlight that oligometastatic cancers have specific molecular profiles, including lower expression of genes associated with aggressive metastatic behavior [17]. These findings underscore the distinct molecular and biological features of oligometastatic disease, which can influence treatment strategies and outcomes.

Furthermore, the response to systemic therapy (e.g., targeted agents or immunotherapy) can further stratify patients, helping to differentiate between indolent oligometastatic disease and occult micrometastatic spread. Biologic markers predictive of oligometastatic behavior remain under investigation but show promising early results. For instance, circulating tumor DNA (ctDNA) has demonstrated potential in identifying minimal residual disease and recurrence risk, although its predictive utility in distinguishing oligometastatic from polymetastatic NSCLC requires further validation in prospective trials. Similarly, mutational profiles involving genes such as ATM, JAK2, MAP2K2, and NTRK1 have been associated with oligometastatic phenotypes in exploratory analyses, suggesting a less aggressive disease course. ATM regulates DNA damage response, and mutation may lead to genomic instability. JAK2 in cytokine signaling may affect immune surveillance. MAP2K2 is part of the MAPK pathway and impacts cell proliferation. Lastly, NTRK1 is involved in neurotrophic signaling.

Since these genes are involved in important cellular signaling pathways, their deregulation could lead to the oligometastatic state and allow for a more regulated and limited pattern of spread [18,19]. However, the current evidence is largely retrospective or observational, and no gene-based classifier has yet been validated from clinical decision making. Continued prospective studies and integrated molecular profiling are needed to establish the predictive value of these biomarkers.

### 2.2. Classification

The classification of OMD has evolved to capture the biological and clinical heterogeneity of this intermediate cancer state (Table 1). Based on the 2023 review by Izmailov et al. [20], OMD can be broadly categorized according to its timing, prior disease history, and behavior under systemic therapy. De novo OMD refers to metastatic disease identified for the first time and is further divided into synchronous (diagnosed within 6 months of the primary tumor) and metachronous (diagnosed after 6 months). Recurrent OMD describes a relapse of oligometastases after prior successful local or systemic treatment. Induced OMD, by contrast, emerges after a history of polymetastatic disease (PMD) that has been controlled by systemic therapy, suggesting a transition back to a limited disease state. Additional subcategories describe disease behavior during therapy: oligorecurrence refers to limited metastases emerging in the absence of active systemic treatment; oligoprogression occurs when only a few metastatic sites progress, while others remain controlled on therapy; and oligopersistence indicates stable limited metastatic disease, persisting despite ongoing systemic treatment. This classification is complemented by clinical and imaging parameters, such as the number of lesions (typically ≤3), number of involved organs, and the lesion size. This definition was established in the landmark randomized trial by Gomez et al. [3] and has been consistently applied in subsequent prospective studies, including LONESTAR [21], NORTHSTAR [22], and BRIGHTSTAR [23], as well as in the 2025 STS Clinical Practice Guidelines. Nevertheless, Dingemans et al. reported a definition of a maximum of five metastases and three organs and excluded diffuse serosal metastases and bone marrow involvement in their definition [24]. Even though no consensus has been reached, these classifications and criteria provide a structured foundation for individualized treatment planning, reflecting the increasingly nuanced understanding of metastatic progression in NSCLC.

### 2.3. Imaging and Staging Considerations

Accurate staging is essential in identifying true oligometastatic disease and avoiding undertreatment or overtreatment. Positron emission tomography–computed tomography (PET/CT) remains the cornerstone of metastatic workup, offering high sensitivity and specificity for extracranial lesions. While PET/CT is highly valuable for extracranial staging, it is not without limitations. False positives can occur due to inflammatory or infectious processes, leading to potential overstaging, and conversely, small-volume metastases or low-metabolic lesions may be missed, resulting in false negatives. These limitations underscore the importance of correlating the PET findings with MRI and the clinical context. In particular, a brain MRI remains essential, as PET has limited sensitivity for detecting intracranial disease [25]. The American Association for Thoracic Surgery (AATS) and the NCCN recommend PET/CT for staging NSCLC due to its ability to provide metabolic information and detect nodal or distant disease [5,26]. However, brain magnetic resonance imaging (MRI) with contrast is mandatory, even in asymptomatic patients, due to the high incidence of brain metastases in NSCLC and the limited sensitivity of PET for intracranial disease. The use of whole-body MRI or advanced functional imaging techniques, such as dual-tracer PET, is still under investigation and not routinely recommended outside of clinical trials.

A multidisciplinary imaging review is recommended to verify the number, location, and resectability of lesions before surgical consideration. In patients with equivocal findings, short-interval follow-up imaging or biopsy may help clarify the true oligometastatic versus polymetastatic biology.

## 3. Role of Surgery in the Management of the Primary Tumor

As previously discussed, surgical resection of the primary lung tumor in patients with OMD has become an area of increasing clinical interest, particularly as evidence accumulates supporting the survival benefit of aggressive local therapy in select patients. Hence, the STS published its first clinical practice guidelines for the management of oligometastatic NSCLC in January 2025. These recommendations reflect a broader shift in the management of metastatic NSCLC.

The STS guideline document provides a Class I recommendation with Level B-R evidence, supporting that surgical resection significantly improves progression-free survival (PFS) and overall survival (OS) when compared to maintenance systemic therapy or best supportive care. This recommendation is based on multiple phase II trials and high-quality retrospective series. Notably, the Gomez et al. randomized trial demonstrated a median PFS of 11.9 months in the LCT arm versus 3.9 months in the observation group and a median OS of 41.2 months vs. 17.0 months [3].

Surgical approaches to the primary tumor in this setting mirror those used for early-stage NSCLC and are dictated by the tumor location, size, and functional status. However, it is pivotal to understand that the oncologic principles for stage IV disease may differ than that of the earlier staged disease, as the primary goal of lobectomy in earlier staged disease is to prevent subsequent metastases. Lobectomy remains the gold standard for most patients with stage II–III resectable primary tumors, offering optimal oncologic control. In well-selected patients with stage I disease, sublobar resection has gained traction, particularly for peripheral lesions or patients with limited pulmonary reserve [27,28]. Regarding the extent of surgical resection in OMD, it is important to acknowledge that the dogma for earlier staged disease may not apply; as such, the guidelines conclude that evidence is currently insufficient to recommend anatomic lobectomy over sublobar resection (segmentectomy or wedge), resulting in a Class IIb recommendation with Level B-NR evidence [14]. The decision regarding lobectomy vs. segmentectomy should be personalized, considering the tumor size and location, pulmonary reserve, anticipated systemic therapy, and the patient’s overall clinical status. 

Likewise, the role of mediastinal lymph node dissection (MLND) in patients undergoing pulmonary resection for OMD in NSCLC remains uncertain, as the benefit may be irrelevant for patients who already have stage IV disease. We acknowledge the prognostic benefit, as well as the notion of removing as much disease as possible. However, there is insufficient evidence to support routine systematic lymphadenectomy in the setting of Stage IV NSCLC (Class IIb recommendation, Level B-NR evidence) [14]. While lymph node involvement (especially pathologic N2 disease) has been associated with worse prognosis across multiple retrospective series [29,30,31,32], there is no conclusive data demonstrating that lymphadenectomy itself improves overall or progression-free survival in stage IV disease. As noted, the studies suggest a prognostic role for nodal status, with better outcomes in pN0 patients compared to those with N1/N2 disease [33]. However, these findings do not necessarily translate into a therapeutic benefit from node removal. As such, lymphadenectomy should be considered on a case-by-case basis, with decisions tailored to the individual risk factors, intraoperative findings, and the goals of surgery, recognizing that nodal sampling may still offer valuable staging and prognostic information. Removal of as much disease as possible should be an important goal that is balanced with the understanding that these are complex operations [34], and patient safety should be the foundation of all intraoperative decision making.

Immune checkpoint inhibitors (ICIs), while pivotal in the management of advanced NSCLC, can induce inflammatory responses and fibrosis in both the tumor and surrounding tissues. These immune-related effects may complicate surgical resection by distorting normal tissue planes, increasing adhesions, and reducing pulmonary compliance. Inflammation-induced fibrosis may also obscure anatomical landmarks, increase the bleeding risk, and prolong the operative time.

However, multiple studies and systematic reviews confirm that surgery after ICI therapy is feasible and generally safe, with perioperative morbidity and mortality rates comparable to those seen after chemotherapy alone. Rates of R0 resection remain high, and the overall complication rates do not appear to be significantly increased, though there was a modest rise in surgery cancellations and high-grade treatment-related adverse events in the ICI group [35,36,37,38].

The most recent phase III data, such as from the perioperative nivolumab trial, show that surgery-related adverse events occur at similar rates in patients receiving neoadjuvant ICI compared to chemotherapy alone. Delays or cancellations of surgery due to adverse events are slightly more frequent with ICI, but the absolute rates remain low [39]. 

## 4. Management of Metastatic Sites

When feasible, surgical resection of metastatic sites in oligometastatic NSCLC can play a critical role in achieving complete local disease control. The rationale for metastasectomy stems from the same principle that guides resection of the primary tumor. Eradicating all known disease in a patient with a limited tumor burden and favorable biology may improve survival and potentially offer a cure. The most commonly resected metastatic sites include the brain and adrenal glands, given their frequency of involvement and accessibility for surgical or ablative treatment.

The body of evidence supporting metastasectomy is most robust for patients with single-organ involvement, especially those with synchronous or metachronous isolated brain or adrenal metastases. Historical series and institutional studies have consistently demonstrated improved outcomes in these patients when surgery is used as part of a multimodal strategy. For example, patients with controlled extracranial disease and surgically resectable brain metastases have been shown to achieve long-term survival when treated with neurosurgical resection or stereotactic radiosurgery in conjunction with systemic therapy [40]. Similarly, adrenalectomy for isolated adrenal metastasis has been associated with favorable survival outcomes, particularly in patients with N0–N1 disease and good performance status [41].

The recent STS 2025 guidelines support surgical resection of metastases as a component of LCT in well-selected patients. Although most randomized trials in this domain have evaluated LCT using radiotherapy, with or without surgery, the principle of addressing all known sites of disease remains central. Notably, the guidelines recommend that surgery be considered even in oligoprogressive or induced oligometastatic disease, provided all disease sites are either controlled or amenable to definitive local therapy. Ultimately, decisions regarding metastasectomy should be individualized and made within a multidisciplinary framework, considering the patient’s response to systemic therapy, the location and number of metastases, and the feasibility of achieving complete resection or local control. Importantly, surgery should never delay systemic therapy or compromise overall treatment sequencing but rather complement it within a coordinated multimodal approach.

## 5. Integration with Systemic Therapy

Systemic therapy remains the cornerstone of treatment in stage IV NSCLC, and its integration with local therapies, including surgery, is essential for optimizing outcomes in patients with OMD. Advances in targeted therapies and ICIs have significantly improved disease control, progression-free survival, and overall survival, particularly for patients with oncogenic driver mutations or high PD-L1 expression.

For patients with oncogenic driver mutations (e.g., EGFR, ALK), targeted therapies such as tyrosine kinase inhibitors (TKIs) have shown superior efficacy compared to chemotherapy. The NCCN guidelines recommend targeted therapies as the initial treatment for patients with advanced NSCLC harboring specific mutations, as these therapies yield higher response rates and are better tolerated [5]. For example, EGFR-TKIs like osimertinib have demonstrated significant improvements in PFS and OS in this patient population [42].

In patients with high PD-L1 expression (≥50%), ICIs such as pembrolizumab have shown substantial benefits. The American Society of Clinical Oncology guidelines recommend PD-1/PD-L1 inhibitors as first-line therapy for these patients, resulting in improved PFS and OS compared to chemotherapy [43]. Studies have shown that adding ICIs to chemotherapy further enhances outcomes, with significant improvements in the PFS and OS [44,45]. Combining systemic therapies with local ablative therapies (LAT), such as surgery or stereotactic body radiotherapy (SBRT), has been shown to provide additional survival benefits. A study demonstrated that patients receiving LAT to all metastatic sites, in conjunction with systemic therapy, had a median OS of 34.4 months and a 5-year OS rate of 37.7% [11]. Another study highlighted that early local therapy combined with PD-1/PD-L1 inhibitors significantly prolonged PFS and OS in oligometastatic NSCLC patients [44]. It is worth mentioning that although single-arm studies combining local therapies with ICIs are promising, they are limited by the lack of controls, and randomized trials are needed to validate the findings.

In alignment with these findings, existing guidelines recommend that surgical resection should be considered after initial systemic therapy in patients who exhibit stable or responsive oligometastatic disease, underscoring the importance of a sequential and coordinated approach [14]. Ultimately, surgery–systemic therapy integration should occur within a multidisciplinary framework, with careful attention to timing, response assessment, and patient fitness.

## 6. Patient Selection and Multidisciplinary Evaluation

Appropriate patient selection is the pivotal key to successful surgical intervention in oligometastatic NSCLC. It is of utmost importance to recognize that only a subset of patients (those with favorable disease biology, adequate performance status, and controlled metastatic burden) derive significant benefit from local therapy. Identifying these candidates requires an integrated assessment of clinical, molecular, and treatment-related factors.

### 6.1. Performance Status, Comorbidities, and Response to Systemic Therapy

Performance status is one of the most important predictors of perioperative risk and overall survival in patients undergoing resection for stage IV NSCLC. Most studies evaluating surgical outcomes in oligometastatic disease restrict inclusion to patients with Eastern Cooperative Oncology Group (ECOG) performance status 0–1 [12].

Equally critical is the response to initial systemic therapy. The most compelling evidence for surgical intervention arises in patients with stable disease or partial response after a course of chemotherapy, targeted therapy, or immunotherapy. This approach allows time to assess the tumor biology and exclude occult polymetastatic progression.

### 6.2. Importance of Multidisciplinary Evaluation

All patients being considered for surgical treatment of oligometastatic NSCLC should undergo formal multidisciplinary evaluation, in many circumstances best achieved through a thoracic oncology tumor board, though practice environments will vary. This approach ensures input from thoracic surgery, medical oncology, radiation oncology, diagnostic radiology, and pathology. Consensus discussion facilitates individualized treatment planning, accurate interpretation of imaging and pathology, and proper sequencing of therapies. Resectability assessment, integration with systemic therapy, and definitive local treatment of all sites should be coordinated to maximize oncologic outcomes while minimizing treatment-related morbidity.

## 7. Outcomes and Prognostic Factors

As previously described, incorporation of surgery into the treatment for oligometastatic NSCLC has been associated with favorable outcomes in carefully selected patients. Both retrospective analyses and prospective clinical trials have demonstrated improved progression-free and overall survival when aggressive local therapy, including surgery, is combined with systemic treatment in patients with a limited metastatic burden.

### 7.1. Survival Data from Retrospective and Prospective Studies

Multiple retrospective studies have reported a median OS ranging from 20 to 40 months in patients undergoing surgery for oligometastatic NSCLC. For example, a study by Deboever et al. reported that patients who underwent pulmonary resection for synchronous oligometastatic NSCLC had a median overall survival of 51.7 months [46]. A phase II trial by De Ruysscher and colleagues demonstrated long-term PFS in patients with synchronous oligometastases treated with radical therapy [47]. A meta-analysis of 757 patients treated with local consolidative therapy (LCT) by Ashworth et al. reported a median OS of 26 months, with a 1-year OS at 70.2% and a 5-year OS at 29.4% [48]. Another study by Mitchell et al. found that pulmonary resection was associated with a median postoperative survival time of 55.2 months, with 1- and 5-year overall survival rates of 95.7% and 48.0%, respectively [12].

Prospective data also support these findings. In the Gomez et al. phase II randomized trial, patients with ≤3 metastases who underwent local consolidative therapy after initial systemic treatment experienced significantly longer progression-free survival (11.9 vs. 3.9 months) compared to those who received maintenance therapy alone. Similar trends have been seen in ongoing trials, which evaluate the combination of systemic therapy with local interventions [21,22,23].

### 7.2. Factors Associated with Prognosis

Key prognostic factors are associated with improved or worse prognosis (Table 2). Factors associated with better outcomes include a limited number of metastases (typically three or fewer), pathologic N0 or N1 nodal status, good performance status (ECOG 0–1), and a sustained response to systemic therapy. Metachronous presentation, as opposed to synchronous disease, is also associated with an improved prognosis, likely reflecting more indolent tumor biology. Molecular features, such as the presence of actionable driver mutations (e.g., *EGFR*, *ALK*), and emerging biomarkers like circulating tumor DNA (ctDNA), may help identify patients most likely to benefit from aggressive local therapy, though prospective validation remains limited.

Conversely, poor prognostic indicators include squamous cell histology, high intrathoracic tumor burden (T3–4), and bone metastases, as noted by Mitchell et al. [49]. A meta-analysis by Li et al. found that aggressive treatment of the primary tumor, female gender, adenocarcinoma histology, absence of nodal disease, and lower (y)pT-stage were associated with improved survival [50]. Genomic profiling is playing an increasingly important role in personalizing therapy. It was found that certain mutations, such as *KRAS* and *SMAD4,* are associated with poor prognosis [51].

## 8. Future Directions

The future of oligometastatic NSCLC treatment will require the integration of data and personalization of treatment for every patient. Additionally, tailoring therapy based on the molecular and genetic characteristics of an individual patient’s tumor will carry importance. Therefore, validation of biomarkers with prospective studies will play a significant role in oligometastatic behavior and treatment response.

Advances in immunotherapy have expanded the systemic treatment setting and are increasingly being combined with local therapies such as stereotactic radiation and surgery. Current guidelines acknowledge the benefits of local consolidative treatment in enhancing survival, but ongoing clinical trials will further refine the timing, sequencing, and patient selection criteria. A growing emphasis on molecular profiling and biomarker development promises to enhance stratification, allowing clinicians to identify which patients are most likely to benefit from aggressive local intervention. As the field evolves, multidisciplinary collaboration will remain essential, ensuring that treatment decisions reflect not only the anatomical staging but also the biological behavior of the disease. Through this combined approach, the management of oligometastatic NSCLC is shifting from a uniform strategy to one that is truly individualized.

In addition to all this, artificial intelligence in imaging interpretation could enhance the detection, staging, and response assessment of oligometastatic NSCLC [52].

## 9. Conclusions

Surgical management of oligometastatic NSCLC represents a significant shift in the treatment paradigm for stage IV disease. Supported by both retrospective and prospective data, surgery offers meaningful survival benefits for well-selected patients when integrated into a multimodal strategy. With the advances in molecular profiling, improved systemic therapies, and increasing recognition of oligometastasis as a distinct biological entity, surgery now plays a definitive role beyond palliation. As ongoing trials continue to improve patient selection and treatment algorithms, a multidisciplinary personalized approach remains essential for maximizing outcomes in this complex and evolving landscape.

## Figures and Tables

**Table 1:** Classification of oligometastatic disease (OMD).

Category	Definition
**De novo OMD**	First presentation of metastatic disease
**Synchronous**	Metastases diagnosed ≤6 months from primary NSCLC
**Metachronous**	Metastases diagnosed >6 months after initial NSCLC diagnosis
**Recurrent OMD**	Relapse of oligometastases after prior successful local/systemic therapy
**Induced OMD**	OMD arising after prior polymetastatic disease controlled by systemic therapy
**Oligorecurrence**	Metastases appearing off treatment
**Oligoprogression**	Limited progression despite overall disease control on systemic therapy
**Oligopersistence**	Persistent limited disease while on systemic therapy

**Table 2:** Key prognostic and selection factors.

Factor	Clinical Implication
**ECOG Performance Status 0–1**	Associated with lower perioperative risk and improved overall survival
**Response to systemic therapy**	Stability or response supports favorable tumor biology and surgical candidacy
**Molecular markers (e.g., EGFR)**	Targetable alterations allow systemic disease control; enable a window for local therapy
**Multidisciplinary consensus**	Ensures appropriate sequencing and coordination of local and systemic treatments
